# Crystal structure, Hirshfeld surface and frontier mol­ecular orbital analysis of 9-(3-bromo-4-hy­droxy-5-meth­oxy­phen­yl)-3,3,6,6-tetra­methyl-3,4,5,6,7,9-hexa­hydro-1*H*-xanthene-1,8(2*H*)-dione

**DOI:** 10.1107/S2056989021010690

**Published:** 2021-10-29

**Authors:** N. Suresh Babu, V. Sughanya, D. Praveenkumar, M. L. Sundararajan

**Affiliations:** a Department of Chemistry, Government College of Engineering-Sengipatti, Thanjavur-613 402, Tamil Nadu, India; b Department of Chemistry, Periyar Government Arts College, Cuddalore-607 001, Tamil Nadu, India; c Department of Chemistry, Swami Vivekananda Arts and Science College, Orathur-605 601, Tamil Nadu, India; d Department of Chemistry, Annamalai University, Annamalai Nagar-608 002, Tamil Nadu, India

**Keywords:** crystal structure, dimedone, xanthene, xanthenedione, pyran ring

## Abstract

In the fused ring system of the title compound, C_24_H_27_BrO_5_, the mean plane and maximum deviations of the central pyran ring are 0.0384 (2) and 0.0733 (2) Å, respectively. The cyclo­hexenone rings both adopt envelope conformations with the tetra-substituted C atoms as flap atoms, whereas the central pyran ring adopts a flattened boat conformation. The central pyran and phenyl substituent rings are almost perpendicular to each other, making a dihedral angle of 89.71 (2)°. In the crystal, pairs of mol­ecules are linked *via* O—H⋯O hydrogen bonds, forming inversion dimers with an *R* ^2^_2_(20) ring motif. A Hirshfeld surface analysis indicates that the most important contributions to the crystal packing are from H⋯H (50.6%), O⋯H/H⋯O (22.9%) and C⋯H/H⋯C (11.1%) contacts. Quantum chemical calculations for the frontier mol­ecular orbitals were undertaken to determine the chemical reactivity of the title compound.

## Abstract 2

In the xanthene moiety of the title compound, the central ring adopts a flattened-boat conformation whereas the cyclo­hexenone rings adopt envelope conformations. In the crystal, mol­ecules are linked by pairs of O—H⋯O hydrogen bonds, forming inversion dimers with an 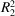 (20) ring motif.

## Chemical context

Xanthene is known as the parent compound of naturally occurring substances with various biological properties including anti­bacterial (Dimmock *et al.*, 1988 ▸), anti­viral (Naidu *et al.*, 2012 ▸), anti­tumor (Al-Omran *et al.*, 2014 ▸) and anti-inflammatory activities (Dimmock *et al.*, 1988 ▸; Cottam *et al.*, 1996 ▸). It is present in organic compounds that are widely used as synthetic dyes (Hilderbrand *et al.*, 2007 ▸), in fluorescent materials used for visualization of biomolecules (Knight *et al.*, 1989 ▸), and in laser technologies (Pohlers *et al.*, 1997 ▸). Ehretianone, a quinonoid xanthene, was reported to possess anti-snake venom activity (Selvanayagam *et al.*, 1996 ▸; Poupelin *et al.*, 1978 ▸). Xanthenes whose structures resemble those of 1,4-di­hydro­pyridines can function as calcium channel blockers (Reddy *et al.*, 2010 ▸; Rathore *et al.*, 2009 ▸). 
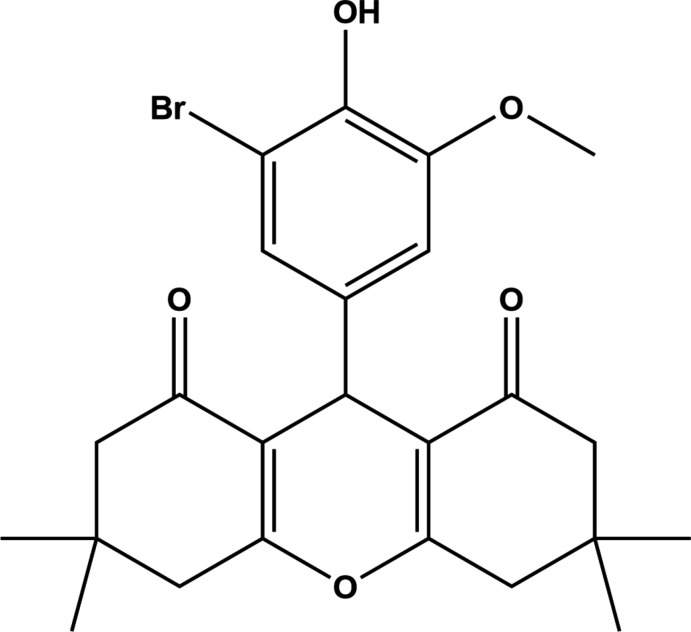


## Structural commentary

**Figure 1:**
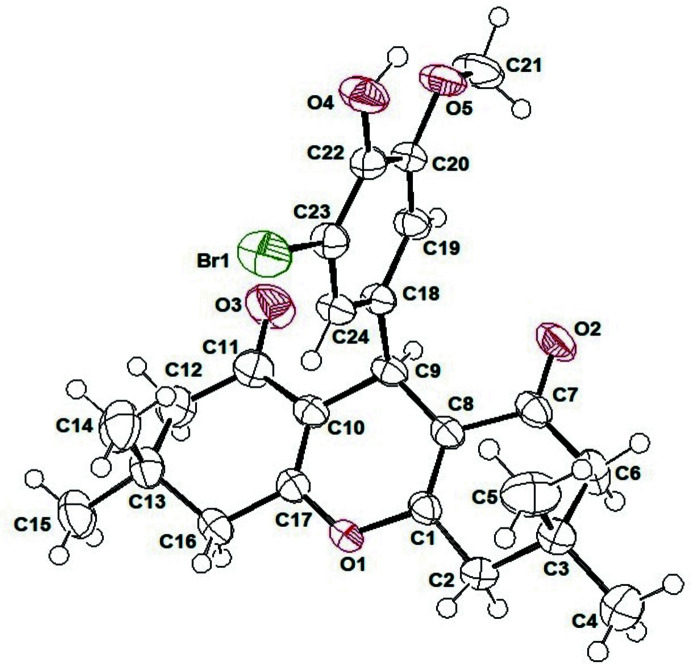
A view of the structure of (I), showing the atom-numbering scheme. Displacement ellipsoids are drawn at the 50% probability level.

The title compound (I) (Fig. 1 ▸) crystallizes in the triclinic space group *P* 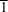 with *Z* = 2. The central pyran ring *B* (O1/C1/C8–C10/C17) is almost planar with a mean deviation from the mean plane of 0.0384 (2) Å and a maximum deviation of 0.0733 (3) Å for C9. Atoms C9 and O1 are displaced out of the mean plane in the the same direction so the ring may also be described as having a highly flattened boat conformation. Both cyclo­hexenone rings, *A* (C10–C13/C16/C17) and *C* (C1–C3/C6–C8), adopt envelope conformations with atoms C13 and C3 as the flaps being situated out of the plane of the ring with deviations of 0.3281 (2) and 0.325 (2) Å, respectively. Rings *A*, *B* and *C* show total puckering amplitudes *Q*(T) of 0.4645 (2), 0.1070 (2) and 0.4607 (16) Å, respectively. The puckering parameters (Cremer & Pople, 1975 ▸) are φ = 179.52 (8)° and θ = 57.55 (2)° for *A*, φ = 178.99 (2)° and θ = 68.92 (2)° for *B*, φ = 304.73 (12)° and θ = 125.47 (2)° for *C*. The planar phenyl substituent and the central pyran ring form a dihedral angle of 89.71 (2)°. In the pyran ring, C1—C8 and C10—C17 are double bonds, as indicated by the bond lengths [C1—C8 = 1.344 (3) Å and C10—C17 = 1.336 (3) Å]. The angles and bond lengths (Allen *et al.*, 1987 ▸; Li *et al.*, 2019 ▸) are within normal ranges. The observed carbonyl bond lengths [C11—O3 = 1.216 (3) and C7—O2 = 1.227 (2) Å] are also normal.

## Supra­molecular features and Hirshfeld surface analysis

**Figure 2:**
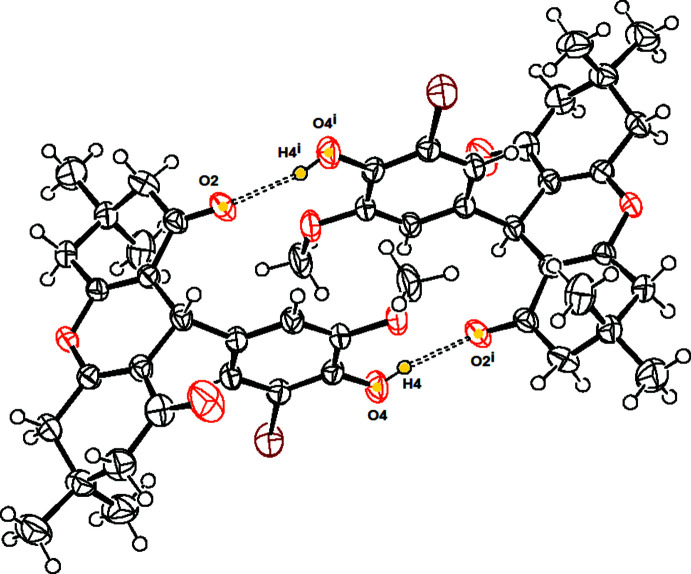
A view of the structure of (I) showing the O—H⋯O hydrogen bonds, forming a centrosymmetric dimer with an 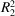 (20) ring motif.

**Figure 3:**
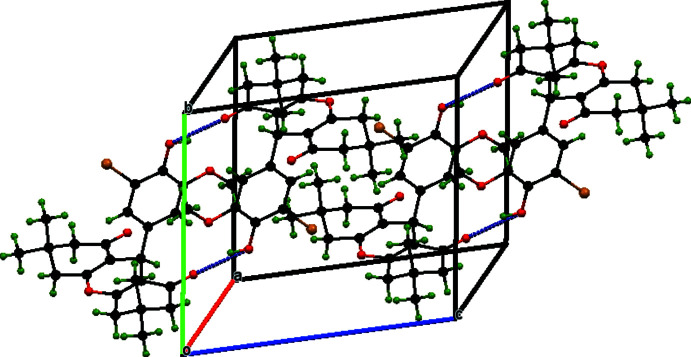
Packing view for (I), showing the formation of O—H⋯O hydrogen bonds between mol­ecules in the unit cell.

In the crystal, mol­ecules are linked by pairs of O4—H4⋯O2 hydrogen bonds (Table 1 ▸), forming inversion dimers with an 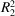 (20) ring motif, parallel to the (001) plane (Fig. 2 ▸). The mol­ecules are further linked by C6—H6*B*⋯O2, C16—H16*A*⋯Br1 and O4—H4⋯O5 hydrogen bonds, forming ribbons (Fig. 3 ▸). Overall, the O—H⋯O and C—H⋯O inter­actions yield a three-dimensional supra­molecular network.

**Figure 4:**
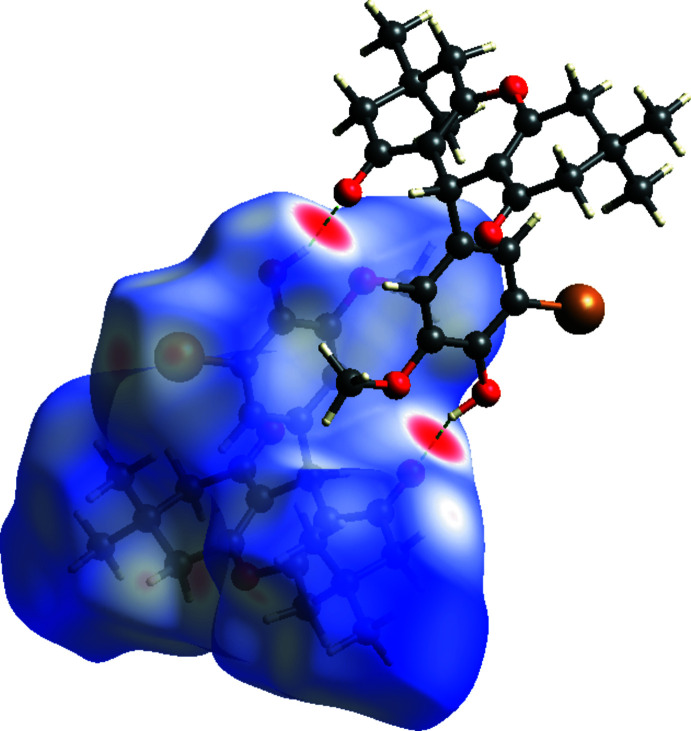
View of the three-dimensional Hirshfeld surface of (I) plotted over *d*_norm_ in the range −0.5451 to 1.6834 a.u. The two O—H⋯O hydrogen bonds forming the dimer are depicted as dashed lines.

**Figure 5:**
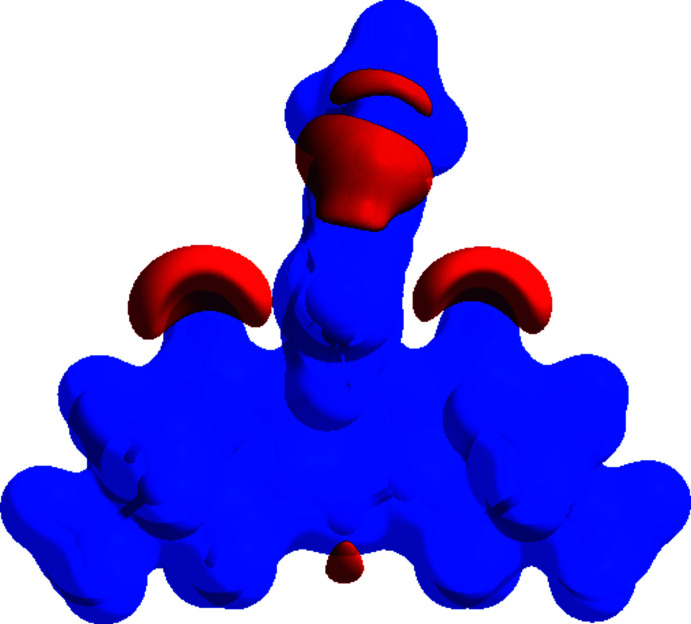
View of the three-dimensional Hirshfeld surface of (I) plotted over electrostatic potential energy in the range −0.0500 to 0.0500 a.u. using the STO-3 G basis set at the Hartree–Fock level of theory. The hydrogen-bond donors and acceptors are viewed as blue and red regions, respectively, around atoms, corresponding to positive and negative potentials.

**Figure 6:**
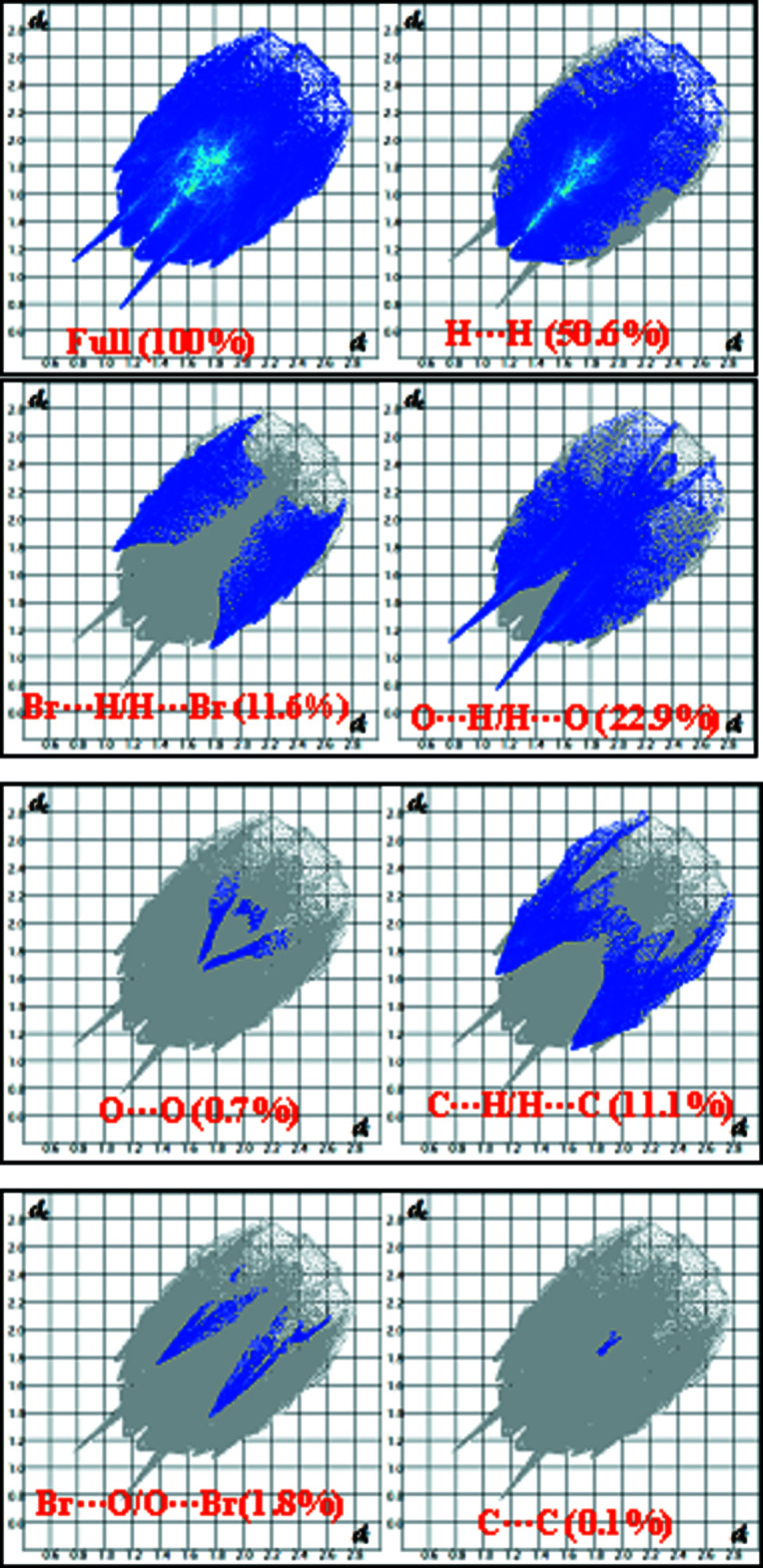
The percentage contributions of close contacts of (I). The *d*_i_ and *d*_e_ values are the closest inter­nal and external distances (in Å) from given points on the Hirshfeld surface.

To qu­antify the inter­molecular contacts in the crystal, Hirshfeld surfaces (Spackman & Jayatilaka, 2009 ▸) and two-dimensional fingerprint plots were generated using *Crystal Explorer 17.5* (Turner *et al.*, 2017 ▸). The Hirshfeld surfaces mapped over *d*_norm_ in the range −0.5451 to 1.6834 a.u. (Fig. 4 ▸) show the inter­molecular contacts as red-coloured spots, which indicate the closer contacts of C—H⋯O and O—H⋯O hydrogen bonds. The bright-red spots indicate their roles as donors and/or acceptors in hydrogen bonding; they also appear as red and blue regions corresponding to negative and positive potentials on the Hirshfeld surface mapped over electrostatic potential (Spackman *et al.*, 2008 ▸) shown in Fig. 5 ▸. Here the red regions indicate negative electrostatic potential (hydrogen-bond acceptors), while the blue regions indicate positive electrostatic potential (hydrogen-bond donors). The 2D fingerprint plots are illustrated in Fig. 6 ▸. The H⋯H contacts comprise 50.6% of the total inter­actions. Besides these contacts, O⋯H/H⋯O (22.9%), C⋯H/H⋯C (11.1%) and Br⋯H/H⋯Br (11.6%) inter­actions make a significant contribution to the total Hirshfeld surface. The percentage contributions of the Br⋯O/O⋯Br, O⋯O and C⋯C contacts are 1.8, 0.7 and 0.1%, respectively.

## Frontier mol­ecular orbital analysis

**Figure 7:**
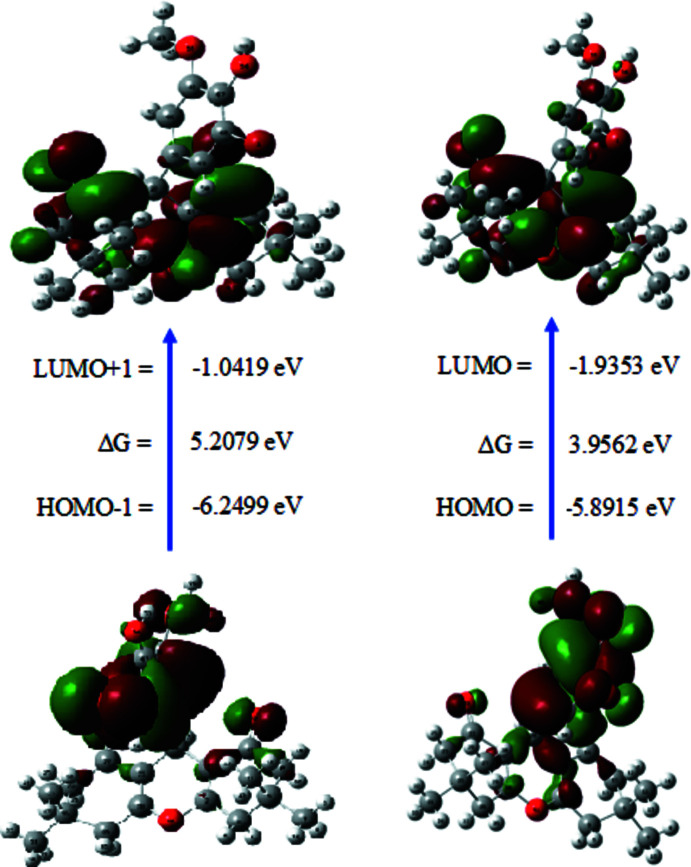
The frontier mol­ecular orbitals of (I).

The chemical reactivity of the title compound was studied by frontier mol­ecular orbital analysis. For the calculation, the starting structural geometry was taken from the refined experimental structure obtained from X-ray diffraction data. The energy levels for the compound were computed using the DFT-B3LYP/6-311G++(d,p) level of theory as implemented in *Gaussian09W* (Frisch *et al.*, 2013 ▸). The calculated frontier mol­ecular orbitals, HOMO, HOMO−1, LUMO and LUMO+1, are shown in Fig. 7 ▸. The energies of HOMO, HOMO−1, LUMO and LUMO+1 were calculated to be −5.8915, −6.2499, −1.9353 and −1.0419 eV, respectively, and the energies required to excite one electron from HOMO to LUMO and from HOMO−1 to LUMO+1 are 3.9562 and 5.2080 eV, respectively. The chemical potential, chemical hardness, chemical softness and electrophilicity index of the title mol­ecule are listed in Table 2 ▸. Parr *et al.* (1999 ▸) have proposed the electrophilicity index as a qu­anti­tative measure of the energy lowering due to the maximal electron flow between donor and acceptor orbitals. The electrophilicity index value of 3.8711 eV shows the global electrophilic nature of the mol­ecule. Based on the wide band gap and its chemical hardness value of 1.9781 eV, the title mol­ecule seems to be hard.

## Database survey

A search of the Cambridge Structural Database (CSD, Version 5.42, update May 2021; Groom *et al.*, 2016 ▸) for the xanthene-1,8(2*H*)-dione unit resulted in 30 hits. They include the following analogues: 2,4-di­nitro­phenyl (LERZEP; Sureshbabu & Sughanya, 2013 ▸), 4-hy­droxy-3,5-di­meth­oxy­phenyl (YAVTAS; Sughanya & Sureshbabu, 2012*a* ▸), 2,4-di­fluoro­phenyl (VITWEC; Fun *et al.*, 2011 ▸), pyridine-2-yl (YIDRIP; Purushothaman & Thiruvenkatam, 2018 ▸). In the title compound, the dihedral angle between the phenyl and pyran rings is 89.71 (2)°, similar to the values observed for LERZEP, the 2,4-di­nitro­phenyl analogue, YAVTAS, the 4-hy­droxy-3,5-di­meth­oxy­phenyl analogue, and VITWEC, the 2,4-di­fluoro­phenyl analogue, for which the dihedral angles are 85.88 (2), 86.32 (2) and 87.55 (4)°, respectively.

## Synthesis and crystallization

Compound (I) was prepared in two stages (Vanag & Stankevich, 1960 ▸). A mixture of 5,5-dimethyl cyclo­hexane-1,3-dione (1.12 g, 8 mmol), 3-bromo-4-hy­droxy-5-meth­oxy­benzaldehyde (0.92 g, 4 mmol) and 20 ml of ethanol were heated to 343 K for about 10 minutes. The reaction mixture was allowed to cool to 298–301 K and the resulting inter­mediate compound, 2,2′-[(3-bromo-4-hy­droxy-5-meth­oxy­phen­yl)methyl­ene]bis­(3-hy­droxy-5,5-di­methyl­cyclo­hex-2-en-1-one) was filtered and dried (m.p. 491 K, 3.4 mmol, yield: 85%) (Sughanya & Sureshbabu, 2012*b* ▸). In the second stage, about 0.50 g (1.04 mmol) of this inter­mediate were dissolved in 20 ml of ethanol. The content was refluxed together with 5 drops of concentrated hydro­chloric acid for 20 minutes with the reaction being monitored by TLC. After completion of the reaction, the reaction mixture was poured into 100 ml of ice-cold water and stirred well. The solid separated was filtered and dried. Yellow single crystals suitable for X-ray diffraction were obtained from 90% ethanol (m.p. 495 K, 0.455 g, 0.96 mmol, yield 92%). IR (KBr): cm^−1^ 3360, 2953, 2865, 1667, 1622, 1584, 1497, 1278, 1234, 1046, 1003. ^1^H NMR (500 MHz, CDCl_3_): 1.04 (*s*, 6H), 1.12 (*s*, 6H), 2.24 (*dd*, *J* = 6 Hz, 4H), 2.47 (*dd*, *J* = 6 Hz, 4H), 3.91 (*s*, 3H), 4.65 (*s*, 1H), 5.88 (*s*, 1H), 6.76 (*s*, 1H), 7.02 (*s*,1H). ^13^C NMR (125 MHz, CDCl_3_): 27.36, 29.21,31.30, 32.23, 40.84, 50.75, 56.32, 107.63, 111.92, 115.26, 123.25, 137.24, 146.56, 162.40, 196.56. ESI–MS: *m*/z: 475.06 [*M* + H].

## Refinement

Crystal data, data collection and structure refinement details are summarized in Table 3 ▸. Hydrogen atoms were fixed geometrically and treated as riding atoms, with C—H = 0.93–0.97 Å and *U*_iso_(H) = 1.2*U*_eq_(C) or 1.5*U*_eq_(C-meth­yl).

## Supplementary Material

Crystal structure: contains datablock(s) global, I. DOI: 10.1107/S2056989021010690/zn2010sup1.cif

Structure factors: contains datablock(s) I. DOI: 10.1107/S2056989021010690/zn2010Isup3.hkl

Supporting information file. DOI: 10.1107/S2056989021010690/zn2010Isup3.cml

CCDC reference: 2064558

Additional supporting information:  crystallographic information; 3D view; checkCIF report

## Figures and Tables

**Table 1:** Hydrogen-bond geometry (Å, °)

*D*—H⋯*A*	*D*—H	H⋯*A*	*D*⋯*A*	*D*—H⋯*A*
C6—H6*B*⋯O2^i^	0.97	2.60	3.377 (3)	137
C16—H16*A*⋯Br1^ii^	0.97	2.94	3.736 (2)	140
O4—H4⋯O2^iii^	0.82	2.04	2.768 (2)	148
O4—H4⋯O5	0.82	2.28	2.701 (2)	113

Symmetry codes: (i) -x+1, -y, -z+2; (ii) -x+1, -y+1, -z+1; (iii) -x+1, -y+1, -z+2.

**Table 2:** The global reactivity descriptors (eV) of the title compound

Frontier mol­ecular orbitals	Energy
*E*_HOMO_	−5.8915
*E*_LUMO_	−1.9353
*E*_HOMO−1_	−6.2499
*E*_LUMO+1_	−1.0419
(*E*_HOMO_ − *E*_LUMO_) gap	3.9562
(*E*_HOMO−1_ − *E*_LUMO+1_) gap	5.2080
Chemical potential (μ)	3.9134
Chemical hardness (η)	1.9781
Chemical softness (*S*)	0.5055
Electrophilicity index (ω)	3.8711

**Table 3:** Experimental details

Crystal data	
Chemical formula	C_24_H_27_BrO_5_
*M*_r_	475.36
Crystal system, space group	Triclinic, *P*\overline{1}
Temperature (K)	296
*a*, *b*, *c* (Å)	9.851 (3), 10.763 (3), 12.313 (3)
α, β, γ (°)	82.38 (1), 66.900 (9), 73.484 (10)
*V* (Å^3^)	1150.9 (5)
*Z*	2
Radiation type	Mo *K*α
μ (mm^−1^)	1.82
Crystal size (mm)	0.30 × 0.25 × 0.20

Data collection	
Diffractometer	Bruker Kappa *APEX3* CMOS
Absorption correction	Multi-scan (*SADABS*; Bruker, 2016 ▸)
*T*_min_, *T*_max_	0.550, 0.746
No. of measured, independent and observed [*I* > 2σ(*I*)] reflections	47600, 4052, 3694
*R*_int_	0.029
(sin θ/λ)_max_ (Å^−1^)	0.595

Refinement	
*R*[*F* ^2^ > 2σ(*F* ^2^)], *wR*(*F* ^2^), *S*	0.027, 0.072, 1.08
No. of reflections	4052
No. of parameters	276
H-atom treatment	H atoms treated by a mixture of independent and constrained refinement
Δρ_max_, Δρ_min_ (e Å^−3^)	0.32, −0.50

Computer programs: *APEX3*, *SAINT-Plus* and *XPREP* (Bruker, 2016 ▸), *SHELXT2018* (Sheldrick, 2015*a* ▸), *SHELXL2018* (Sheldrick, 2015*b* ▸), *ORTEP-3 for Windows* (Farrugia, 2012 ▸), *Mercury* (Macrae *et al.*, 2020 ▸) and *publCIF* (Westrip, 2010 ▸).

## supplementary crystallographic information

### Crystal data

**Table:**

C_24_H_27_BrO_5_	*F*(000) = 492
*M**_r_* = 475.36	*D*_x_ = 1.372 Mg m^−^^3^
Triclinic, *P*1	Melting point: 495 K
*a* = 9.851 (3) Å	Mo *K*α radiation, λ = 0.71073 Å
*b* = 10.763 (3) Å	Cell parameters from 9325 reflections
*c* = 12.313 (3) Å	θ = 2.6–30.0°
α = 82.38 (1)°	µ = 1.82 mm^−^^1^
β = 66.900 (9)°	*T* = 296 K
γ = 73.484 (10)°	BLOCK, yellow
*V* = 1150.9 (5) Å^3^	0.30 × 0.25 × 0.20 mm
*Z* = 2	

### Data collection

**Table:**

Bruker Kappa APEX3 CMOS diffractometer	4052 independent reflections
Radiation source: fine-focus sealed tube	3694 reflections with *I* > 2σ(*I*)
Graphite monochromator	*R*_int_ = 0.029
ω and φ scan	θ_max_ = 25.0°, θ_min_ = 3.4°
Absorption correction: multi-scan (SADABS; Bruker, 2016)	*h* = −11→11
*T*_min_ = 0.550, *T*_max_ = 0.746	*k* = −12→12
47600 measured reflections	*l* = −14→14

### Refinement

**Table:**

Refinement on *F*^2^	0 restraints
Least-squares matrix: full	Hydrogen site location: mixed
*R*[*F*^2^ > 2σ(*F*^2^)] = 0.027	H atoms treated by a mixture of independent and constrained refinement
*wR*(*F*^2^) = 0.072	*w* = 1/[σ^2^(*F*_o_^2^) + (0.0296*P*)^2^ + 0.6561*P*] where *P* = (*F*_o_^2^ + 2*F*_c_^2^)/3
*S* = 1.08	(Δ/σ)_max_ = 0.001
4052 reflections	Δρ_max_ = 0.32 e Å^−^^3^
276 parameters	Δρ_min_ = −0.50 e Å^−^^3^

### Special details

**Table:**

Geometry. All esds (except the esd in the dihedral angle between two l.s. planes) are estimated using the full covariance matrix. The cell esds are taken into account individually in the estimation of esds in distances, angles and torsion angles; correlations between esds in cell parameters are only used when they are defined by crystal symmetry. An approximate (isotropic) treatment of cell esds is used for estimating esds involving l.s. planes.

### Fractional atomic coordinates and isotropic or equivalent isotropic displacement parameters (Å^2^)

**Table:**

	*x*	*y*	*z*	*U*_iso_*/*U*_eq_	
Br1	0.49132 (3)	0.69406 (2)	0.62363 (2)	0.05292 (10)	
C1	0.5946 (2)	0.11911 (17)	0.64639 (15)	0.0319 (4)	
C2	0.4569 (2)	0.0735 (2)	0.66746 (17)	0.0412 (5)	
H2A	0.484623	−0.020439	0.666673	0.049*	
H2B	0.420396	0.105743	0.603872	0.049*	
C3	0.3280 (2)	0.1192 (2)	0.78588 (18)	0.0423 (5)	
C4	0.2057 (3)	0.0452 (3)	0.8150 (2)	0.0676 (7)	
H4A	0.250419	−0.046032	0.819885	0.101*	
H4B	0.125530	0.073181	0.889224	0.101*	
H4C	0.164507	0.062167	0.754031	0.101*	
C5	0.2564 (3)	0.2644 (2)	0.7781 (3)	0.0617 (7)	
H5A	0.332860	0.310996	0.759881	0.092*	
H5B	0.215134	0.281231	0.717207	0.092*	
H5C	0.176157	0.292245	0.852400	0.092*	
C6	0.3982 (3)	0.0887 (2)	0.88067 (18)	0.0516 (6)	
H6A	0.320679	0.124356	0.954643	0.062*	
H6B	0.427417	−0.004661	0.891856	0.062*	
C7	0.5345 (2)	0.13965 (17)	0.85383 (16)	0.0366 (4)	
C8	0.6339 (2)	0.15206 (16)	0.72946 (15)	0.0313 (4)	
C9	0.7704 (2)	0.20575 (17)	0.70112 (16)	0.0322 (4)	
C10	0.8672 (2)	0.19047 (17)	0.57072 (16)	0.0322 (4)	
C11	1.0196 (2)	0.2146 (2)	0.52605 (19)	0.0439 (5)	
C12	1.1113 (2)	0.2068 (2)	0.3948 (2)	0.0495 (5)	
H12A	1.180310	0.121046	0.378089	0.059*	
H12B	1.172975	0.268803	0.372815	0.059*	
C13	1.0158 (2)	0.2333 (2)	0.31752 (18)	0.0428 (5)	
C14	0.9298 (3)	0.3771 (2)	0.3217 (2)	0.0617 (7)	
H14A	1.001793	0.429336	0.291989	0.093*	
H14B	0.869236	0.392418	0.273983	0.093*	
H14C	0.864478	0.399531	0.401812	0.093*	
C15	1.1185 (3)	0.1973 (3)	0.1891 (2)	0.0627 (7)	
H15A	1.189481	0.250422	0.158058	0.094*	
H15B	1.173617	0.107698	0.186491	0.094*	
H15C	1.056780	0.211083	0.142489	0.094*	
C16	0.9043 (2)	0.14713 (19)	0.36435 (16)	0.0378 (4)	
H16A	0.831875	0.172240	0.325133	0.045*	
H16B	0.960435	0.057845	0.345098	0.045*	
C17	0.8192 (2)	0.15590 (17)	0.49441 (15)	0.0313 (4)	
C18	0.7174 (2)	0.34791 (17)	0.73598 (16)	0.0318 (4)	
C19	0.7345 (2)	0.38397 (18)	0.83369 (16)	0.0342 (4)	
H19	0.786711	0.322584	0.873846	0.041*	
C20	0.6744 (2)	0.51052 (18)	0.87108 (16)	0.0346 (4)	
C21	0.7625 (3)	0.4676 (3)	1.0299 (2)	0.0604 (7)	
H21A	0.759056	0.510392	1.094887	0.091*	
H21B	0.716974	0.395977	1.059224	0.091*	
H21C	0.866680	0.436440	0.977932	0.091*	
C22	0.5972 (2)	0.60496 (17)	0.81104 (16)	0.0341 (4)	
C23	0.5878 (2)	0.56797 (18)	0.71128 (16)	0.0334 (4)	
C24	0.6451 (2)	0.44079 (18)	0.67421 (16)	0.0345 (4)	
H24	0.634765	0.418239	0.608017	0.041*	
O1	0.68252 (15)	0.12340 (13)	0.52771 (10)	0.0355 (3)	
O2	0.56680 (19)	0.16747 (15)	0.93260 (12)	0.0493 (4)	
O3	1.0717 (2)	0.2361 (2)	0.59419 (16)	0.0743 (6)	
O4	0.53332 (19)	0.72988 (13)	0.84657 (13)	0.0476 (4)	
H4	0.531593	0.735147	0.912952	0.071*	
O5	0.68156 (19)	0.55546 (14)	0.96789 (13)	0.0489 (4)	
H9	0.829 (2)	0.156 (2)	0.7488 (18)	0.035 (5)*	

### Atomic displacement parameters (Å^2^)

**Table:**

	*U*^11^	*U*^22^	*U*^33^	*U*^12^	*U*^13^	*U*^23^
Br1	0.06708 (17)	0.03847 (13)	0.05825 (15)	0.00106 (10)	−0.03853 (12)	−0.00177 (9)
C1	0.0423 (10)	0.0251 (9)	0.0255 (9)	−0.0070 (8)	−0.0102 (8)	−0.0021 (7)
C2	0.0519 (12)	0.0401 (11)	0.0348 (10)	−0.0190 (9)	−0.0126 (9)	−0.0071 (8)
C3	0.0471 (12)	0.0427 (11)	0.0369 (10)	−0.0158 (9)	−0.0104 (9)	−0.0078 (9)
C4	0.0628 (16)	0.0841 (19)	0.0579 (15)	−0.0401 (15)	−0.0067 (13)	−0.0113 (14)
C5	0.0558 (14)	0.0518 (14)	0.0809 (18)	−0.0008 (11)	−0.0323 (13)	−0.0200 (13)
C6	0.0582 (14)	0.0608 (14)	0.0293 (10)	−0.0206 (11)	−0.0070 (10)	0.0027 (10)
C7	0.0520 (12)	0.0273 (9)	0.0273 (9)	−0.0037 (8)	−0.0157 (9)	−0.0007 (7)
C8	0.0428 (10)	0.0230 (8)	0.0262 (9)	−0.0039 (7)	−0.0138 (8)	−0.0010 (7)
C9	0.0409 (10)	0.0287 (9)	0.0294 (9)	−0.0032 (8)	−0.0191 (8)	−0.0027 (7)
C10	0.0371 (10)	0.0267 (9)	0.0319 (9)	−0.0021 (7)	−0.0153 (8)	−0.0036 (7)
C11	0.0407 (11)	0.0464 (12)	0.0470 (12)	−0.0073 (9)	−0.0206 (10)	−0.0034 (9)
C12	0.0385 (11)	0.0577 (14)	0.0491 (12)	−0.0127 (10)	−0.0115 (10)	−0.0049 (10)
C13	0.0417 (11)	0.0446 (11)	0.0349 (10)	−0.0099 (9)	−0.0080 (9)	0.0006 (9)
C14	0.0723 (17)	0.0451 (13)	0.0584 (15)	−0.0140 (12)	−0.0192 (13)	0.0114 (11)
C15	0.0563 (14)	0.0825 (18)	0.0388 (12)	−0.0235 (13)	−0.0026 (11)	−0.0026 (12)
C16	0.0416 (11)	0.0396 (10)	0.0286 (9)	−0.0056 (8)	−0.0115 (8)	−0.0050 (8)
C17	0.0345 (10)	0.0265 (9)	0.0300 (9)	−0.0038 (7)	−0.0116 (8)	−0.0017 (7)
C18	0.0361 (10)	0.0308 (9)	0.0305 (9)	−0.0077 (8)	−0.0138 (8)	−0.0049 (7)
C19	0.0412 (10)	0.0338 (10)	0.0328 (9)	−0.0091 (8)	−0.0194 (8)	−0.0009 (8)
C20	0.0432 (11)	0.0373 (10)	0.0287 (9)	−0.0155 (8)	−0.0140 (8)	−0.0050 (8)
C21	0.0862 (18)	0.0673 (16)	0.0484 (13)	−0.0213 (14)	−0.0426 (13)	−0.0100 (11)
C22	0.0376 (10)	0.0295 (9)	0.0347 (10)	−0.0097 (8)	−0.0104 (8)	−0.0060 (8)
C23	0.0361 (10)	0.0318 (9)	0.0346 (10)	−0.0061 (8)	−0.0175 (8)	−0.0001 (8)
C24	0.0415 (10)	0.0345 (10)	0.0317 (9)	−0.0076 (8)	−0.0178 (8)	−0.0062 (8)
O1	0.0420 (7)	0.0434 (7)	0.0240 (6)	−0.0152 (6)	−0.0112 (5)	−0.0044 (5)
O2	0.0715 (10)	0.0505 (9)	0.0272 (7)	−0.0142 (8)	−0.0204 (7)	−0.0024 (6)
O3	0.0588 (11)	0.1243 (17)	0.0587 (11)	−0.0374 (11)	−0.0297 (9)	−0.0097 (11)
O4	0.0675 (10)	0.0325 (7)	0.0435 (8)	−0.0032 (7)	−0.0248 (8)	−0.0116 (6)
O5	0.0739 (10)	0.0411 (8)	0.0421 (8)	−0.0138 (7)	−0.0303 (8)	−0.0103 (6)

### Geometric parameters (Å, º)

**Table:**

Br1—C23	1.8954 (19)	C12—H12A	0.9700
C1—C8	1.344 (3)	C12—H12B	0.9700
C1—O1	1.378 (2)	C13—C16	1.530 (3)
C1—C2	1.489 (3)	C13—C14	1.535 (3)
C2—C3	1.537 (3)	C13—C15	1.536 (3)
C2—H2A	0.9700	C14—H14A	0.9600
C2—H2B	0.9700	C14—H14B	0.9600
C3—C5	1.529 (3)	C14—H14C	0.9600
C3—C4	1.530 (3)	C15—H15A	0.9600
C3—C6	1.534 (3)	C15—H15B	0.9600
C4—H4A	0.9600	C15—H15C	0.9600
C4—H4B	0.9600	C16—C17	1.489 (3)
C4—H4C	0.9600	C16—H16A	0.9700
C5—H5A	0.9600	C16—H16B	0.9700
C5—H5B	0.9600	C17—O1	1.378 (2)
C5—H5C	0.9600	C18—C24	1.380 (3)
C6—C7	1.494 (3)	C18—C19	1.395 (2)
C6—H6A	0.9700	C19—C20	1.383 (3)
C6—H6B	0.9700	C19—H19	0.9300
C7—O2	1.227 (2)	C20—O5	1.378 (2)
C7—C8	1.471 (3)	C20—C22	1.400 (3)
C8—C9	1.511 (3)	C21—O5	1.403 (3)
C9—C10	1.515 (3)	C21—H21A	0.9600
C9—C18	1.530 (2)	C21—H21B	0.9600
C9—H9	0.98 (2)	C21—H21C	0.9600
C10—C17	1.336 (3)	C22—O4	1.362 (2)
C10—C11	1.470 (3)	C22—C23	1.384 (3)
C11—O3	1.216 (3)	C23—C24	1.387 (3)
C11—C12	1.510 (3)	C24—H24	0.9300
C12—C13	1.533 (3)	O4—H4	0.8200
			
C8—C1—O1	122.25 (17)	H12A—C12—H12B	107.6
C8—C1—C2	126.24 (17)	C16—C13—C12	107.75 (17)
O1—C1—C2	111.51 (15)	C16—C13—C14	110.89 (18)
C1—C2—C3	112.21 (16)	C12—C13—C14	110.14 (19)
C1—C2—H2A	109.2	C16—C13—C15	108.20 (18)
C3—C2—H2A	109.2	C12—C13—C15	110.44 (19)
C1—C2—H2B	109.2	C14—C13—C15	109.39 (19)
C3—C2—H2B	109.2	C13—C14—H14A	109.5
H2A—C2—H2B	107.9	C13—C14—H14B	109.5
C5—C3—C4	109.3 (2)	H14A—C14—H14B	109.5
C5—C3—C6	111.04 (18)	C13—C14—H14C	109.5
C4—C3—C6	109.72 (19)	H14A—C14—H14C	109.5
C5—C3—C2	109.94 (19)	H14B—C14—H14C	109.5
C4—C3—C2	109.43 (17)	C13—C15—H15A	109.5
C6—C3—C2	107.36 (18)	C13—C15—H15B	109.5
C3—C4—H4A	109.5	H15A—C15—H15B	109.5
C3—C4—H4B	109.5	C13—C15—H15C	109.5
H4A—C4—H4B	109.5	H15A—C15—H15C	109.5
C3—C4—H4C	109.5	H15B—C15—H15C	109.5
H4A—C4—H4C	109.5	C17—C16—C13	112.68 (16)
H4B—C4—H4C	109.5	C17—C16—H16A	109.1
C3—C5—H5A	109.5	C13—C16—H16A	109.1
C3—C5—H5B	109.5	C17—C16—H16B	109.1
H5A—C5—H5B	109.5	C13—C16—H16B	109.1
C3—C5—H5C	109.5	H16A—C16—H16B	107.8
H5A—C5—H5C	109.5	C10—C17—O1	123.42 (16)
H5B—C5—H5C	109.5	C10—C17—C16	125.17 (18)
C7—C6—C3	115.04 (17)	O1—C17—C16	111.41 (15)
C7—C6—H6A	108.5	C24—C18—C19	119.37 (17)
C3—C6—H6A	108.5	C24—C18—C9	119.97 (16)
C7—C6—H6B	108.5	C19—C18—C9	120.58 (16)
C3—C6—H6B	108.5	C20—C19—C18	120.33 (17)
H6A—C6—H6B	107.5	C20—C19—H19	119.8
O2—C7—C8	119.91 (19)	C18—C19—H19	119.8
O2—C7—C6	121.64 (18)	O5—C20—C19	124.98 (18)
C8—C7—C6	118.42 (17)	O5—C20—C22	114.18 (16)
C1—C8—C7	117.75 (18)	C19—C20—C22	120.84 (17)
C1—C8—C9	123.31 (16)	O5—C21—H21A	109.5
C7—C8—C9	118.90 (16)	O5—C21—H21B	109.5
C8—C9—C10	109.09 (15)	H21A—C21—H21B	109.5
C8—C9—C18	110.02 (15)	O5—C21—H21C	109.5
C10—C9—C18	111.38 (15)	H21A—C21—H21C	109.5
C8—C9—H9	107.8 (12)	H21B—C21—H21C	109.5
C10—C9—H9	110.4 (11)	O4—C22—C23	119.68 (17)
C18—C9—H9	108.0 (12)	O4—C22—C20	122.66 (17)
C17—C10—C11	118.59 (17)	C23—C22—C20	117.67 (16)
C17—C10—C9	122.45 (17)	C22—C23—C24	121.99 (17)
C11—C10—C9	118.96 (16)	C22—C23—Br1	119.16 (14)
O3—C11—C10	120.3 (2)	C24—C23—Br1	118.84 (14)
O3—C11—C12	121.2 (2)	C18—C24—C23	119.71 (16)
C10—C11—C12	118.47 (18)	C18—C24—H24	120.1
C11—C12—C13	114.73 (18)	C23—C24—H24	120.1
C11—C12—H12A	108.6	C1—O1—C17	118.39 (14)
C13—C12—H12A	108.6	C22—O4—H4	109.5
C11—C12—H12B	108.6	C20—O5—C21	117.51 (16)
C13—C12—H12B	108.6		
			
C8—C1—C2—C3	24.0 (3)	C12—C13—C16—C17	49.6 (2)
O1—C1—C2—C3	−156.46 (16)	C14—C13—C16—C17	−71.0 (2)
C1—C2—C3—C5	72.5 (2)	C15—C13—C16—C17	169.04 (18)
C1—C2—C3—C4	−167.4 (2)	C11—C10—C17—O1	175.94 (16)
C1—C2—C3—C6	−48.4 (2)	C9—C10—C17—O1	−4.1 (3)
C5—C3—C6—C7	−67.4 (2)	C11—C10—C17—C16	−4.0 (3)
C4—C3—C6—C7	171.63 (19)	C9—C10—C17—C16	175.98 (17)
C2—C3—C6—C7	52.8 (2)	C13—C16—C17—C10	−24.5 (3)
C3—C6—C7—O2	151.79 (19)	C13—C16—C17—O1	155.55 (16)
C3—C6—C7—C8	−30.3 (3)	C8—C9—C18—C24	−68.8 (2)
O1—C1—C8—C7	−177.95 (16)	C10—C9—C18—C24	52.3 (2)
C2—C1—C8—C7	1.5 (3)	C8—C9—C18—C19	107.97 (19)
O1—C1—C8—C9	4.6 (3)	C10—C9—C18—C19	−130.94 (18)
C2—C1—C8—C9	−175.96 (17)	C24—C18—C19—C20	2.5 (3)
O2—C7—C8—C1	179.28 (17)	C9—C18—C19—C20	−174.21 (17)
C6—C7—C8—C1	1.3 (3)	C18—C19—C20—O5	178.32 (18)
O2—C7—C8—C9	−3.1 (3)	C18—C19—C20—C22	−0.9 (3)
C6—C7—C8—C9	178.92 (17)	O5—C20—C22—O4	−0.8 (3)
C1—C8—C9—C10	−10.9 (2)	C19—C20—C22—O4	178.46 (18)
C7—C8—C9—C10	171.59 (15)	O5—C20—C22—C23	178.72 (17)
C1—C8—C9—C18	111.52 (19)	C19—C20—C22—C23	−2.0 (3)
C7—C8—C9—C18	−65.9 (2)	O4—C22—C23—C24	−177.13 (18)
C8—C9—C10—C17	10.7 (2)	C20—C22—C23—C24	3.3 (3)
C18—C9—C10—C17	−111.0 (2)	O4—C22—C23—Br1	2.0 (3)
C8—C9—C10—C11	−169.39 (16)	C20—C22—C23—Br1	−177.59 (14)
C18—C9—C10—C11	69.0 (2)	C19—C18—C24—C23	−1.3 (3)
C17—C10—C11—O3	−174.2 (2)	C9—C18—C24—C23	175.50 (17)
C9—C10—C11—O3	5.9 (3)	C22—C23—C24—C18	−1.7 (3)
C17—C10—C11—C12	3.5 (3)	Br1—C23—C24—C18	179.19 (14)
C9—C10—C11—C12	−176.41 (18)	C8—C1—O1—C17	3.6 (2)
O3—C11—C12—C13	−156.7 (2)	C2—C1—O1—C17	−175.94 (15)
C10—C11—C12—C13	25.6 (3)	C10—C17—O1—C1	−3.8 (3)
C11—C12—C13—C16	−50.9 (2)	C16—C17—O1—C1	176.09 (15)
C11—C12—C13—C14	70.2 (2)	C19—C20—O5—C21	3.2 (3)
C11—C12—C13—C15	−168.9 (2)	C22—C20—O5—C21	−177.50 (19)

### Hydrogen-bond geometry (Å, º)

**Table:**

*D*—H···*A*	*D*—H	H···*A*	*D*···*A*	*D*—H···*A*
C6—H6*B*···O2^i^	0.97	2.60	3.377 (3)	137
C16—H16*A*···Br1^ii^	0.97	2.94	3.736 (2)	140
O4—H4···O2^iii^	0.82	2.04	2.768 (2)	148
O4—H4···O5	0.82	2.28	2.701 (2)	113

Symmetry codes: (i) −*x*+1, −*y*, −*z*+2; (ii) −*x*+1, −*y*+1, −*z*+1; (iii) −*x*+1, −*y*+1, −*z*+2.

### The frontier molecular orbital energies of the title compound

**Table:**

Orbitals	a.u	eV
V_130_	-0.00040	-0.01088
V_129_	-0.00433	-0.11782
V_128_	-0.00548	-0.14911
V_127_	-0.00823	-0.22394
V_126_	-0.01615	-0.43945
V_125_	-0.03829	-1.04190
V_124_	-0.07112	1.93524
O_123_	-0.21651	-5.89145
O_122_	-0.22968	-6.24982
O_121_	-0.24696	-6.72002
O_120_	-0.25386	-6.90778
O_119_	-0.25681	-6.98805
O_118_	-0.28020	-7.62452
O_117_	-0.28631	-7.79078
O_116_	-0.29688	-8.07840
O_115_	-0.33387	-9.08493
O_114_	-0.33908	-9.22670

* O- Occupied orbital V- Vacant orbital a.u-atomic unit eV-Electron Volt
